# Fabrication of Tri-Directional Poly(2,5-benzimidazole) Membrane Using Direct Casting for Vanadium Redox Flow Battery

**DOI:** 10.3390/polym15173577

**Published:** 2023-08-28

**Authors:** Jung-Kyu Jang, Tae-Ho Kim

**Affiliations:** Hydrogen Energy Research Center, Korea Research Institute of Chemical Technology (KRICT), Daejeon 34114, Republic of Korea

**Keywords:** vanadium redox flow battery, PBI membrane, poly(2,5-benzimidazole), ABPBI, direct casting method

## Abstract

In vanadium redox flow batteries (VRFBs), simultaneously achieving high proton conductivity, low vanadium-ion permeability, and outstanding chemical stability using electrolyte membranes is a significant challenge. In this study, we report the fabrication of a tri-directional poly(2,5-benzimidazole) (T-ABPBI) membrane using a direct casting method. The direct-cast T-ABPBI (D-T-ABPBI) membrane was fabricated by modifying the microstructure of the membrane while retaining the chemical structure of ABPBI, having outstanding chemical stability. The D-T-ABPBI membrane exhibited lower crystallinity and an expanded free volume compared to the general solvent-cast T-ABPBI (S-T-ABPBI) membrane, resulting in enhanced hydrophilic absorption capabilities. Compared to the S-T-ABPBI membrane, the enhanced hydrophilic absorption capability of the D-T-ABPBI membrane resulted in a decrease in the specific resistance (the area-specific resistance of S-T-ABPBI and D-T-ABPBI membrane is 1.75 and 0.98 Ωcm^2^, respectively). Additionally, the D-T-ABPBI membrane showed lower vanadium permeability (3.40 × 10^−7^ cm^2^ min^−1^) compared to that of Nafion 115 (5.20 × 10^−7^ cm^2^ min^−1^) due to the Donnan exclusion effect. Owing to the synergistic effects of these properties, the VRFB assembled with D-T-ABPBI membrane had higher or equivalent coulomb efficiencies (>97%) and energy efficiencies (70–91%) than Nafion 115 at various current densities (200–40 mA cm^−2^). Furthermore, the D-T-ABPBI membrane exhibited stable performance for over 300 cycles at 100 mA cm^−2^, suggesting its outstanding chemical stability against the highly oxidizing VO_2_^+^ ions during practical VRFB operation. These results indicate that the newly fabricated D-T-ABPBI membranes are promising candidates for VRFB application.

## 1. Introduction

Renewable energy sources, such as solar and wind energy, are promising sources of sustainable and eco-friendly energy. Although renewable energy sources can supply electrical energy, such sources are unpredictable and unstable, and their output fluctuates with changes in the Earth’s climate. Therefore, the energy storage system (ESS) that can enable the storage of electric energy obtained from renewable sources and its on-demand release is required for the reliable and stable implementation of renewable energy in electric-grid applications [1,2]. Various types of battery-based ESS, including Li-ion [3] and Li-selenium batteries [4], as well as Zn-ion batteries [5] and others [6], have been continuously under development up to the present time. Among the ESS studied to date, the vanadium redox flow battery (VRFB) has emerged as a potential candidate for use as a large-scale RFB owing to its unique advantages, such as a long lifespan and high safety scalability [7,8].

Ion-exchange membranes (IEMs), as the core components of VRFBs, enable the separation of positive and negative electrolytes and the conduction of protons between both electrolytes. Perfluorosulfonic acid membranes, also known as Nafion, are widely used as IEMs in VRFBs owing to their high proton conductivity and outstanding chemical stability. However, using Nafion in VRFB systems has some limitations, such as high costs and vanadium crossover [9,10]. Therefore, non-fluorinated cation/anion exchange membranes based on hydrocarbon polymers have been studied to replace Nafion. However, some challenges persist, such as poor chemical stability in highly oxidizing vanadium electrolytes [11,12,13].

Recently, acid-doped polybenzimidazole (PBI) membranes have emerged as promising candidates for the IEMs of VRFBs because of their low vanadium crossover, high selectivity, low cost, and outstanding VRFB cycling stability [14,15,16]. Based on these advantages, PBI membranes, including porous [15,17,18] and dense membranes with chemical modification [19,20,21], have been widely studied for VRFBs. The main objective of these studies has been to improve conductivity by developing an ion-transport pathway to improve the absorption of acid and water while suppressing the crossover of vanadium as much as possible [22].

Among the PBI-family materials, poly(2,5-benzimidazole) (ABPBI) has the simplest structure, comprising only a benzimidazole group in a head-to-tail sequence. ABPBI has the most basic –N= site per polymer chain, which can form hydrogen bonds or participate in acid–base interactions with acid molecules. In addition, ABPBI offers many advantages, such as low cost and outstanding thermal and mechanical properties [23]. In particular, our previous study reported that ABPBI has superior chemical stability in highly oxidizing VO_2_^+^ solutions when compared with that of commercial PBI (i.e., m-PBI) [24].

However, some challenges are associated with using ABPBI as an electrolyte membrane for VRFBs. First, ABPBI membranes must be fabricated using strong acids (such as methanesulfonic acid) instead of common organic solvents because of their extremely poor solubility in the latter [25]. This makes their manufacturing process hazardous and challenging. Second, ABPBI membranes exhibit lower conductivities (higher area-specific resistance) than those of other PBI membranes [26] after being doped in a 3–5 M sulfuric acid solution, which is similar to the concentration of the sulfuric-acid solution used in the electrolyte of VRFBs. ABPBI polymers form strong hydrogen bonds between the chains owing to their structural features. As a consequence, the ABPBI membrane is formed with densely packed polymer chains during the process of solvent evaporation. This is anticipated to result in an ABPBI membrane with a small free volume. For this reason, the density of the ABPBI membrane is known to be higher than that of other PBI membranes (the density of ABPBI and m-PBI is 1.5 and 1.33 g/cm^3^, respectively) [25]. This causes the ABPBI membrane to insufficiently absorb acid and water molecules despite having many basic –N= sites per polymer chain. Previously, we reported that acid and water absorption could be enhanced by suppressing polymer-chain packing by introducing an alkyl side group into ABPBI with controlled graft ratios [24]. The alkyl side-group-grafted ABPBI membrane with an appropriate ratio exhibited a significantly higher energy efficiency (EE) than that of pristine ABPBI membranes. This result indicates that hindering the chain packing of the ABPBI polymer segments is an effective way to increase the free volume of the membrane and consequently enhance its acid- and water-absorption capabilities. However, as more alkyl side groups are grafted onto the ABPBI polymer, the oxidative stability deteriorates because of the change in the electron density of the grafted benzimidazole group.

**Scheme 1:**
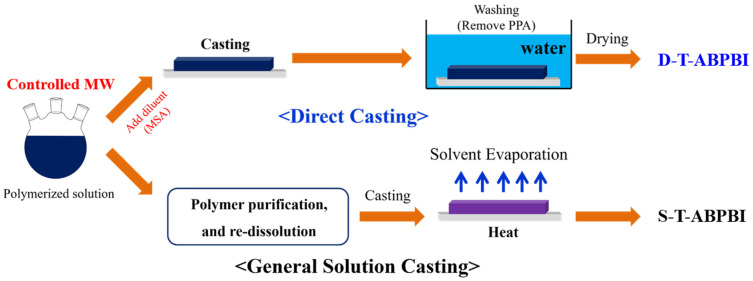
Schematic of direct casting and general solution casting of PBI membranes.

Based on this observation, we propose the fabrication of an ABPBI-derivative membrane via a direct casting process. A direct-cast ABPBI-derivative membrane can be fabricated without modifying the chemical structure of the ABPBI polymer. Therefore, the membrane can maintain outstanding chemical stability in the oxidizing environment of the VRFB electrolyte. In the direct casting method, the polymerized solution is directly spread on a substrate, without polymer purification, after polymerization has sufficiently proceeded (as shown in Scheme 1). Subsequently, the cast-polymerized solution is continuously exposed to controlled humidity. In this state, polyphosphoric acid (PPA), which is used as a polymerization solvent, is hydrolyzed, forming a solidified PBI membrane in the sol–gel state [27,28,29]. The direct casting of PBI membranes is a time- and cost-saving environmentally friendly technique. Previous studies have demonstrated that PBI membranes formed via direct casting have an amorphous structure with suppressed polymer-chain packing [30,31], resulting in an increased free volume of the PBI membrane. Given the abovementioned advantages, in this study, the physicochemical and electrochemical properties of the direct-cast ABPBI-derivative membranes were studied. Single-cell VRFBs with direct-cast ABPBI-derivative membranes were tested for performance and stability, and the results were compared with those of Nafion 115.

## 2. Materials and Methods

### 2.1. Materials

For this study, 3,4-diaminobenzoic acid (DABA, >98%) was purchased from TCI (Tokyo, Japan) and used without purification. 1,3,5-Benzenetricarboxylic acid (BTCA, 95%) and methaesulfonic acid (MSA) were obtained from Aldrich (St. Louis, MO, USA). BTCA was purified via recrystallization in distilled water. PPA (116%) was purchased from Junsei Chemicals (Tokyo, Japan). Sulfuric acid (95–97%) was purchased from Merck (Darmstadt, Germany), and sodium bicarbonate was obtained from Duksan Pure Chemicals Ltd. (Seoul, Republic of Korea).

### 2.2. Polymer Synthesis and Membrane Preparation

**Figure 1:**
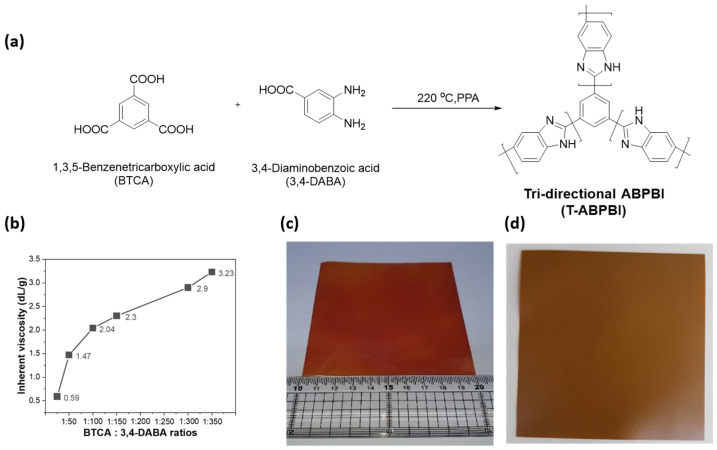
(**a**) Synthesis of T-ABPBI, (**b**) inherent viscosity of T-ABPBI based on ratios of BTCA and 3,4-DABA, and photographs of (**c**) D-T-ABPBI and (**d**) S-T-ABPBI membranes.

Tri-directional ABPBIs (T-ABPBIs) were synthesized via the condensation polymerization of 3,4-DABA and BTCA at various mole ratios in PPA, as previously reported (see Figure 1a) [32]. The total solid content of the initial reaction solution was 10 wt%. The molar ratios of 3,4-DABA and BTCA were adjusted to control the molecular weight of T-ABPBIs. The detailed procedure for the synthesis of T-ABPBI, with a 3,4-DABA–BTCA molar ratio of 250:1, is as follows: BTCA (0.03 g, 0.143 mmol) and PPA (49.14 g) were added to a 250-mL four-neck reactor fitted with a mechanical stirrer, nitrogen inlet, and calcium chloride drying tube. The reactor was heated to 140 °C for 30 min to dissolve BTCA. Next, 3,4-DABA (5.43 g, 35.690 mmol) was gradually added over 3 h, and stirring was continued for 1 h. Subsequently, the solution was heated to 220 °C, and the temperature was maintained for 2 h to ensure the completion of the condensation reaction. During this time, the mixture became a viscous dark brown solution. After the completion of the reaction, the temperature was decreased to room temperature.

The membranes were fabricated using two methods: (1) direct casting and (2) general solution casting. The procedure for fabricating the membrane via direct casting was as follows: After cooling, the polymerized solution was diluted with MSA to obtain a solid content of 5% and stirred for an additional 24 h before direct casting. The diluted solution, at 30 °C, was spread on a quartz glass plate and was evenly flattened using a film applicator. Thereafter, the cast solution was immersed in a water bath at 25 °C to obtain a solidified membrane. The membrane was washed several times with deionized water to remove the PPA. The wet membrane was dried on a vacuum plate for 12 h to remove water before being stored in a desiccator. The membrane formed via direct casting is denoted as the D-T-ABPBI membrane.

The synthesis process for general solution casting was as follows: After cooling the polymerized solution, the reaction mixture was poured into distilled water (1 dL) to precipitate the polymer before filtration. The polymer was neutralized with 5 wt% sodium bicarbonate aqueous solution (1 dL) and washed several times with distilled water. The T-ABPBI polymer powder was further washed with a 5% sodium bicarbonate aqueous solution (300 mL) in a Soxhlet extractor for 2 d, followed by drying under vacuum conditions at 80 °C for 48 h. The T-ABPBI powder (0.8 g) was dissolved in MSA (25 mL) at 50 °C. The polymer solution was filtered through a glass filter and spread onto a clean quartz plate, followed by heating to 135 °C for 24 h to evaporate the MSA. After cooling to room temperature, the T-ABPBI membrane was peeled off the quartz plate by immersing it in a water bath. The membrane was rinsed with water for 24 h to eliminate traces of residual MSA. The membrane was dried on a vacuum plate for 12 h to remove water before being stored in a desiccator. The membrane fabricated via general solvent casting is denoted as the S-T-ABPBI membrane.

### 2.3. Polymer Characterization

The inherent viscosity (IV) of the polymers polymerized with various ratios of two monomers was measured in 95–97% sulfuric acid (0.05 dL g^−1^) at 25 °C using a viscometer (Cannon Ubbelohde). ^1^H-Nuclear magnetic resonance (NMR) spectra of the polymers were recorded using a spectrometer (500 MHz; Bruker, Billerica, MA, USA). Deuterated sulfuric acid-d2 was used as the NMR solvent, and its chemical shifts (11.5 ppm) were used as a reference. The structures of the fabricated membranes were confirmed via attenuated total reflectance-infrared (ATR-IR) spectroscopy (ALPHA-T FT-IR spectrometer, Bruker, Billerica, MA, USA).

### 2.4. Membrane Characterization

Wide-angle X-ray diffraction (WAXD) patterns of the membranes (without acid doping) were measured using Cu Kα radiation (λ = 1.54 Å) with a wide-angle X-ray diffractometer (D/MAX-2200, Rigaku, Tokyo, Japan). The measurements were performed in the range of 5–40°. The membranes were dried at 80 °C in a vacuum for 24 h before the WAXD patterns were measured. A precision impedance analyzer (4924A, Agilent, Santa Clara, CA, USA) was used to determine the dielectric constants. Parallel-plate capacitors were manufactured to measure the dielectric properties. A gold electrode was fabricated via sputtering on both sides of the resulting coin-type films (19.65 mm^2^).

Hydrophilic-absorption capability of the membranes was measured using previously reported methods [19]. The acid-doping process for the membranes was performed in an acid bath containing sulfuric acid at a concentration equivalent to that of the vanadium electrolyte. The weights (*W*_undoped_) of the specimens were measured before immersion in the 4 M H_2_SO_4_ solution at room temperature for 2 d. The acid-doped specimens (which had absorbed H_2_SO_4_ and water) were wiped lightly with Kimwipes, and their weights (*W*_wet_) were measured again. Finally, the specimens were dried at 80 °C in a vacuum for 2 d to remove water, and their weights (*W*_doped_) were measured. Acid (*W_A_*) and water (*W_W_*) uptakes were calculated using the following equations:

(1) $$W_{A}(wt\%)=\left[{(W}_{doped}-W_{undoped}\right)/W_{undoped}]\times 100$$

(2) $$W_{W}(wt\%)=\left[{(W}_{wet}-W_{doped}\right)/W_{undoped}]\times 100$$

The area-specific resistance (ASR) of the membranes was measured in a 4 M H_2_SO_4_ solution using an impedance analyzer equipped with a two-probe clip cell. Prior to the ASR measurements, the samples were immersed in a 4 M H_2_SO_4_ solution for 2 d at room temperature. The ASRs of the tested membranes were calculated using the following equation:

(3) $$R=\left(R_{1}-R_{2}\right)\times S$$

where *R*_1_ and *R*_2_ are the electrical resistances of the cells with and without the membrane, respectively, and *S* is the effective area (0.196 cm^2^) of the two-probe clip cell.

The permeabilities of the fabricated membranes to vanadium species were measured using a previously described method [19]. The VO^2+^ ion permeability of the membranes was tested using a diffusion cell. Membranes with an effective area of 2.86 cm^2^ were positioned between the two cells. The cells on the right and left were filled with 2 M MgSO_4_ in a 3 M H_2_SO_4_ (80 mL) solution and 2 M VOSO_4_ in a 3 M H_2_SO_4_ (80 mL) solution, respectively. The sulfuric acid concentration, 3 M, of each test solution was selected to prevent the precipitation of V(IV) ions during the measurement. Samples were periodically obtained from the right cell, and the VO^2+^ ion concentration was measured using a UV–vis spectrometer (Cary 8454, Agilent Technologies, Santa Clara, CA, USA). The permeability of the membranes to VO^2+^ ions was calculated using the following equation:

(4) $$V_{R}\frac{dC_{R}\left(t\right)}{dt}=A\frac{P}{L}\left(C_{L}-C_{R}\left(t\right)\right)$$

where *V*_R_ is the volume of the solution in the cell on the right; *C*_L_ is the VO^2+^ ion concentration in the cell on the left; *C*_R_(*t*) represents the VO^2+^ ion concentration in the cell on the right side as a function of time (*t*); *A* and *L* represent the effective area and thickness of the membrane, respectively; and *P* is the VO^2+^ permeability of the membrane.

### 2.5. VRFB Single-Cell Test

A VRFB single cell was fabricated by placing a membrane between two carbon-felt electrodes secured with two graphite bipolar plates. Two copper plates were used as current collectors. The effective membrane area was 7 cm × 7 cm (49 cm^2^). Both the positive and negative electrolytes contained 80 mL of 1.65 M V^3+^/VO^2+^ in 4 M H_2_SO_4_. The performance of the VRFB single cell was evaluated using a battery cycler (857 redox cell test system, Scribner Associates Inc., Southern Pines, NC, USA) at current densities ranging from 200 to 40 mAcm^−2^. The rate of electrolyte flow was 100 mL min^−1^, and the cut-off voltage was set between 1.0 and 1.7 V. Furthermore, the charge–discharge cycling test of the single-cell VRFB was performed for 300 cycles at 100 mAcm^−2^. The voltage efficiency (VE), Columbic efficiency (CE), EE, and electrolyte utilization (EU) of the charging–discharging cycling process were calculated as follows:

(5) $$VE=\frac{EE}{CE}\times 100\%$$

(6) $$CE=\frac{\int I_{d}dt}{\int I_{c}dt}\times 100\%$$

(7) $$EE=\frac{\int V_{d}I_{d}dt}{\int V_{c}I_{c}dt}\times 100$$

(8) $$EU=\frac{C_{d-real}}{C_{d-theoretical}}\times 100\%$$

where *I*, *V*, and *t* represent the current, voltage, and time, respectively; subscripts c and d denote the charge and discharge processes, respectively; C_d-real_ and C_d-theoretical_ are, respectively, the real and theoretical discharge capacities (3537 mAh), calculated based on the amount of redox-active materials.

## 3. Results

### 3.1. Polymer Synthesis and Membrane Preparation

The molar ratios of 3,4-DABA and BTCA were adjusted to control the viscosity of the polymerized solution (i.e., control the molecular weight of the polymer) to make it suitable for the direct casting method. Generally, obtaining high-molecular-weight ABPBI is simple because 3,4-DABA ensures a 100% stoichiometric ratio. However, the viscosity of the polymerizing solution can suddenly increase, resulting in a solution with a high viscosity at some point during polymerization. As shown in Figure 1b, T-ABPBIs were synthesized by varying the BTCA-to-DABA molar ratio from 1:25 to 1:350. The inherent viscosity of T-ABPBI increased as the ratio of 3,4-DABA increased. The inherent viscosities of the T-ABPBIs synthesized at BTCA-to-DABA molar ratios of 1:50, 1:100, 1:150, and 1:300 were 1.47, 2.04, 2.30, and 2.9 dL/g, respectively. The optimal inherent viscosity of T-ABPBIs was between 2.30 and 2.90 dL/g for the direct casting method with a 10 wt% solid content. When the inherent viscosity of T-ABPBI was less than 2 dL/g, the direct-cast membranes exhibited poor mechanical properties. T-ABPBI, having an inherent viscosity of 2.9 dL/g, exhibited a tensile strength exceeding 80 MPa, suggesting excellent mechanical properties, as shown in ESI (Figure S1). However, when the viscosity was greater than 3 dL/g, the viscosity of the polymerized solution was too high, making it difficult to handle. Figure 1c,d show images of direct-cast T-ABPBI (D-T-ABPBI) and general-solvent-cast T-ABPBI (S-T-ABPBI) membranes, respectively. The D-T-ABPBI membranes were fabricated over a large area (100 cm^2^). In addition, these membranes were more transparent than the S-T-ABPBI membrane, suggesting that the D-T-ABPBI membranes had lower crystallinity. Additionally, the fabricated membranes exhibited an apparently dense and compact morphology as shown in ESI (Figure S2).

### 3.2. Wide-Angle X-ray Diffraction and Dielectric Constant

The crystallinities of the fabricated membranes were confirmed by measuring their WAXD patterns. The densely ordered regions in the crystalline structure reduced the free volume between polymer chains [33,34]. Therefore, the penetration of water and acid molecules through a membrane with a crystalline region is expected to be difficult after doping with an acidic aqueous solution, owing to its small free volume. In the application of a PBI-based membrane in a VRFB, the absorption of acid and water molecules into the PBI membrane significantly affects ion conduction within the membranes [22].

**Figure 2:**
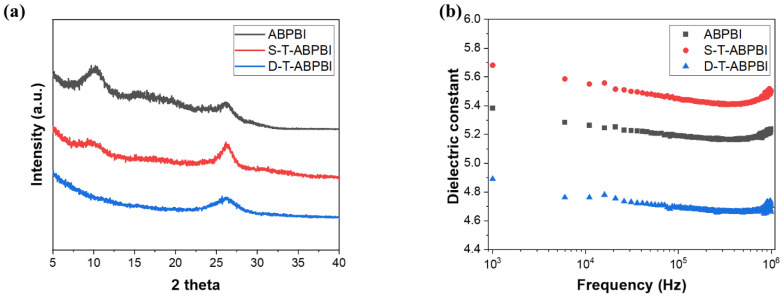
(**a**) Wide-angle X-ray diffraction patterns and (**b**) dielectric constant measurements for ABPBI, S-T-ABPBI, and D-T-ABPBI membranes.

In Figure 2a, ABPBI exhibits two peaks at 11° and 26°, indicating the presence of two different types of crystal structures. The peak at 26° originates from the parallel orientation of the polymer chains dominated by the H-bonded region [31], and the peak at 11° indicates looser chain packing in the weakly H-bonded region [35]. S-T-ABPBI also shows two different peaks at 11° and 26°, indicating that the crystalline structure of S-T-ABPBI is similar to that of ABPBI. However, unlike the degree of crystallinity of ABPBI, the degree of crystallinity of S-T-ABPBI at 26° was stronger than that at 11°. The calculated *d*_sp_ values at 11° and 26° were 8.0 Å and 3.42 Å, respectively. The WAXD pattern indicates that the crystallinity of the S-T-ABPBI membranes is dominated by closer chain packing when compared with that of ABPBI. The D-T-ABPBI membranes show only one peak at 26°, indicating that crystallinity is attributable only to the π-π interaction and parallel orientation of the polymer chain. Hence, the D-T-ABPBI membranes were subjected to crystalline changes during the direct casting process. The WAXD patterns of the fabricated membranes exhibited various crystal structures; however, this does not fully explain the change in the free volume of the membranes caused by the induced change in the crystal structure.

The dielectric constants (ε) of the membranes were measured to assess their relative free volume based on the changes in crystallinity. The dielectric constant of a polymer is primarily affected by two factors: (1) the free volume of the membrane and (2) the number of polarizable groups per unit volume [36]. In this study, the contribution of the number of polarizable groups was considered equivalent because all the polymers comprised only the benzimidazole group. Therefore, changes in the dielectric constant were mainly reflected in the contribution of the free volume. An increase in the free volume of the membrane decreased the dielectric constant. As shown in Figure 2b, the dielectric constant of the S-T-ABPBI membranes was higher than that of the ABPBI membrane, indicating a smaller free volume of the membrane compared with that of the ABPBI membrane. As shown by the WAXD results, S-T-ABPBI had a closer chain-packing structure when compared with that of ABPBI, and a smaller free volume was expected. In contrast, the D-T-ABPBI membrane exhibited the lowest dielectric constant, suggesting that D-T-ABPBI had the largest free volume among the tested membranes. Even though a parallel orientation of the polymer chain still existed in the D-T-ABPBI membrane after direct casting, the free volume of the D-T-ABPBI membrane may have increased owing to the decrease in crystallinity in the looser chain packing of the weak H-bonded region. We hypothesize that the expanded free volume after direct casting enhanced the hydrophilic absorption capability of the D-T-ABPBI membrane, as discussed in Section 3.3.

### 3.3. Absorption and Ion-Conduction Properties of PBI Membranes

**Figure 3:**
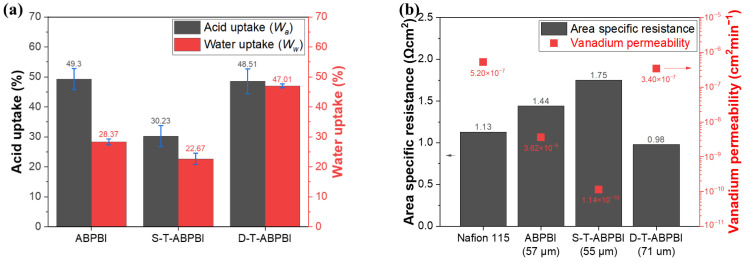
(**a**) Acid (*W_A_*) and water (*W_W_*) uptakes of fabricated membranes. (**b**) Area-specific resistance and vanadium permeability of Nafion 115, S-T-ABPBI, and D-T-ABPBI membranes.

The absorption behaviors of the tested membranes in 4 M H_2_SO_4_ are shown in Figure 3a. Even though the membranes had almost the same chemical structure, consisting mostly of benzimidazole repeating units, their absorption capabilities differed when doped with 4 M H_2_SO_4_. This indicates that the free volume of the membranes, which is related to their crystallinity, affects their absorption capabilities when doped with a 4 M H_2_SO_4_ solution. As shown in Figure 3a, S-T-ABPBI exhibited the lowest *W_A_* and *W_W_* among all the samples, and ABPBI membranes exhibited higher *W_A_* and *W_W_* than that of the S-T-ABPBI membrane (*W_A_* = 49.3% for ABPBI and 30.2% for S-T-ABPBI; *W_W_* = 28.4% for ABPBI and 22.7% for S-T-ABPBI). The difference in absorption capabilities can be explained using the dissimilarity in the relative free volume. It is difficult for water and acid molecules to penetrate the S-T-ABPBI membrane because it has a smaller free volume compared with that of the ABPBI membrane. Similarly, the D-T-ABPBI membrane, which had the largest free volume among the fabricated membranes, showed a superior hydrophilic absorption capability than that of the other membranes (*W_A_* = 48.5% and *W_W_* = 47.0% for D-T-ABPBI). Generally, the proton conduction of dense PBI membranes for VRFB applications is highly dependent on their acid and water absorption capabilities [22,37,38]. Therefore, a D-T-ABPBI membrane with enhanced *W_A_* and *W_W_* is expected to exhibit better proton conduction compared with that of other PBI membranes, as discussed below.

The ASR values of all membranes were measured in 4 M H_2_SO_4_. Figure 3b presents a comparison of the ASR values of the Nafion 115 (141 μm), ABPBI (57 μm), S-T-ABPBI (55 μm), and D-T-ABPBI (71 μm) membranes. The thicknesses of the fabricated membranes were measured after doping with a 4 M H_2_SO_4_ solution. The ASR of Nafion 115 was as low as 1.13 Ωcm^2^, indicating its outstanding proton-conducting capability. In contrast, S-T-ABPBI exhibited the highest ASR (1.75 Ωcm^2^) among the fabricated membranes despite its thinness. Its ASR was more than 1.5 times higher than that of Nafion 115. As mentioned previously, the high ASR of the S-T-ABPBI membrane is hypothesized to originate from the insufficient absorption of acid and water molecules because of its relatively small free volume. The ABPBI membrane, which had the same thickness as that of the S-T-ABPBI membrane, showed a lower ASR (1.44 Ωcm^2^) than that of the S-T-ABPBI membrane owing to enhanced hydrophilic absorption capability. Notably, the D-T-ABPBI membrane, which was thicker than the ABPBI and S-T-ABPBI membranes, showed a remarkably low AR of only 0.98 Ωcm^2^. This result confirms that the expanded free volume of the PBI membrane effectively improves its proton-conducting ability by enhancing its acid- and water-absorption capabilities. In addition, the ASR of the D-T-ABPBI membrane was as low as that of Nafion 115, indicating excellent proton conduction.

The electrolyte membrane in a VRFB must be designed to prevent vanadium crossover, which reduces the self-discharge rate and maintains a high CE. Figure 3b shows the permeability of the membranes to vanadium ions. The vanadium permeability of Nafion 115 was close to the reported value (5.20 × 10^−7^ cm^2^ min^−1^) [26,39], and the ABPBI and S-T-ABPBI membranes exhibited considerably lower vanadium permeabilities (3.62 × 10^−9^ and 1.14 × 10^−10^ cm^2^ min^−1^, respectively) compared with that of Nafion 115. In VRFB electrolytes containing 3–5 M H_2_SO_4_, the imidazole group of PBI becomes positively charged via protonation by acid molecules. This leads to electrostatic repulsion (Donnan exclusion effect) between the positively charged imidazole group of the PBI membrane and positive vanadium ions, resulting in an extremely low vanadium crossover [22,40]. Even though the D-T-ABPBI membrane exhibited a higher vanadium permeability (3.40 × 10^−7^ cm^2^ min^−1^) compared with that of the other PBI membranes, its vanadium permeability was still lower than that of Nafion 115. Despite the Donnan effect acting between the protonated D-T-ABPBI membranes and vanadium ions, an increase in the amount of water filling the expanded free volume is considered to have promoted the crossover of vanadium ions [41].

Considering both the ASR and vanadium permeability, the D-T-ABPBI membrane exhibits a balance between high proton conduction and a vanadium crossover lower than that of Nafion 115.

### 3.4. VRFB Single-Cell Test and Long-Term Cycling Stability

**Figure 4:**
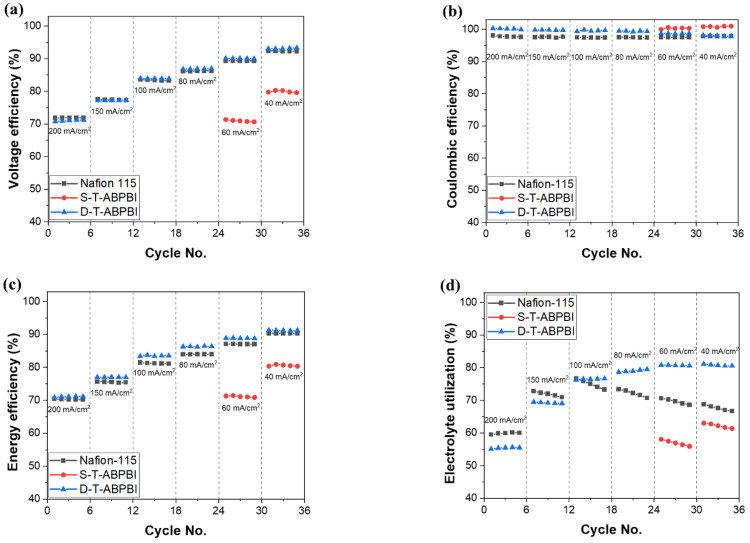
VRFB single-cell performances of Nafion 115, S-T-ABPBI, and D-T-ABPBI membranes at various current densities (200–40 mA cm^−2^): (**a**) voltage efficiency, (**b**) Coulombic efficiency, (**c**) energy efficiency, and (**d**) electrolyte utilization.

Figure 4 shows the single-cell performances of the membranes tested at various current densities. The test was continuously performed from high to low current density (from 200 to 40 mA cm^−2^) to minimize the decay of efficiencies owing to electrolyte crossover. As shown in Figure 4a, the VEs of all membranes increased with a decrease in current density owing to the decreased Ohmic polarization. For the S-T-ABPBI membrane, cells could only be measured at current densities below 60 mA cm^−2^ and exhibited a significantly lower VE than those of other cells. The inability of the S-T-ABPBI membrane to operate at high current densities was evident, owing to the high ASR induced by its low absorption capabilities and crystallinity. In contrast, the D-T-ABPB cell showed VEs similar to those of Nafion 115 at all current densities, indicating the outstanding proton conduction of D-T-ABPBI and Nafion 115.

As seen in Figure 4b, the single cells assembled with the S-T-ABPBI membrane exhibited CEs above 99% at low current densities (60 and 40 mA cm^−2^). The extremely low VO^2+^ permeability of the S-T-ABPBI membrane contributed to the CE of the S-T-ABPBI membrane. However, despite its high CE value, the S-T-ABPBI membrane is unsuitable as an electrolyte membrane in VRFBs owing to its insufficient proton conductivity (i.e., low VE) across all current densities. Nafion 115 exhibited CE values of 97–98% at all current densities. The CE of Nafion 115 should decrease with a decrease in current density because of the longer crossover time of the vanadium species. However, the electrolyte imbalance caused by electrolyte transfer that occurs in the continuous charging–discharging process decreases the crossover time of the vanadium species in the electrolyte, increasing the CE. The CE of the D-T-ABPBI membrane gradually decreased from 99.9% to 97.8% as the current density decreased from 200 to 40 mA cm^−2^, which was slightly higher than that of Nafion 115. As described in the preceding section, the vanadium permeability of the D-T-membrane was marginally lower than that of the Nafion 115 membrane because of the Donnan effect induced by the protonated PBI, resulting in a slightly higher CE of the D-T-membrane than that of the Nafion 115 membrane.

The EE, which is the product of VE and CE, is an important factor to consider when assessing energy loss during the charge–discharge process. Nafion 115 is known for its high efficiency over a wide range of test current densities compared with other Nafions of various thicknesses [9], indicating that it is the most suitable membrane for VRFB applications. The EE of the S-T-ABPBI membrane is more than 10% lower than that of Nafion 115, even at low current densities. In contrast, D-T-ABPBI exhibited an EE equal to or higher than that of the Nafion 115 membrane at all current densities because of the combination effect of its outstanding CE and VE (Figure 4c).

The EU was calculated for the Nafion 115, S-T-ABPBI, and D-TABPBI membranes based on the theoretical discharge capacity. The primary factors affecting the EU are vanadium crossover and Ohmic polarization [39]. As depicted in Figure 4d, the EU of the D-T-ABPBI membrane was lower than that of the Nafion 115 membrane at current densities above 150 mA cm^−2^, primarily owing to the marginally higher Ohmic polarization. However, D-T-ABPBI exhibited higher EU at current densities below 100 mA cm^−2^, suggesting a well-balanced combination of VE and CE in the D-T-ABPBI membrane. Notably, the EU of the D-T-ABPBI membrane remained relatively stable for five cycles at the same current density, indicating minimal electrolyte transfer. In contrast, the Nafion 115 membrane exhibited a noticeable decrease in EU as the charging–discharging process progressed at the same current density. Consequently, the EU of the Nafion 115 membrane continued to decline throughout the testing period, whereas that of the D-T-ABPBI membrane remained stable. These results confirm that the D-T-ABPBI membrane effectively suppresses vanadium-ion crossover during the charging–discharging process.

**Figure 5:**
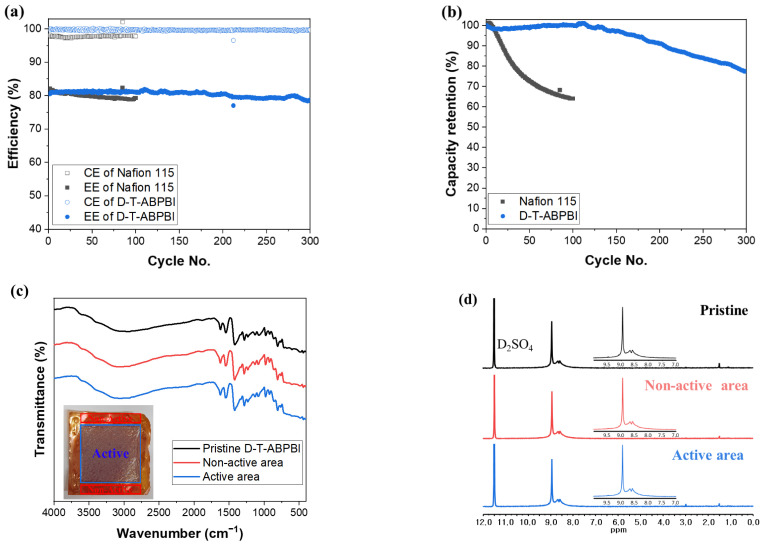
Long-term cycling test results of VRFB single cells with Nafion 115 and D-T-ABPBI membranes at 100 mA cm^−2^: (**a**) efficiency, (**b**) discharge-capacity retention, (**c**) ATR-IR spectra, and (**d**) ^1^H-NMR (in D_2_SO_4_) of fabricated membranes after in situ test (after 300 cycles at 100 mA cm^−2^).

A long-term cycling test was performed on the fabricated membranes at a current density of 100 mA cm^−2^, as depicted in Figure 5a. Throughout the test period, the D-T-ABPBI membrane consistently exhibited a CE of more than 99% for 300 cycles without any fluctuation, surpassing that of the Nafion 115 cell (97–98%). Moreover, during the long-term battery-cycling test, no rapid drop in efficiency resulting from membrane degradation was observed for the D-T-ABPBI membrane. Although the initial EE of the D-T-ABPBI membrane was marginally lower than that of Nafion 115, it surpassed the latter after 20 cycles. In contrast, the EE of the Nafion 115 membrane decreased continuously because of an imbalance in the electrolyte caused by vanadium crossover during the test period.

Figure 5b shows the capacity retentions of the tested membranes. The cell assembled with the Nafion 115 membrane exhibited a rapid decline in capacity, reaching 65% capacity retention after 100 cycles, which resulted in the termination of the Nafion 115 cycling test. As frequently mentioned, VRFBs assembled with Nafion membranes experience significant deterioration in their capacity [10]. This capacity decay primarily occurs due to a substantial crossover of vanadium species through the membrane. In contrast, the cell assembled with the D-T-ABPBI membrane exhibited stable capacity retention for up to 150 cycles, followed by a gradual decrease. We believe that the relatively slow capacity decrease in VRFBs assembled with the D-TABPBI membrane is due to the inhibited cross of vanadium ions caused by the Donnan effect. For the effective operation of actual VRFBs, suppressing the capacity decline caused by electrolyte crossover is crucial to minimize the capital costs of operation and electrolyte rebalancing.

The chemical stability of the D-T-ABPBI membrane was analyzed after the long-term cycling tests. Figure 5c shows the ATR-IR spectra of the pristine membrane and the non-active and active areas of the tested D-T-ABPBI membrane. The ATR-IR spectra of the non-active area of the tested membranes did not exhibit any significant differences, suggesting minimal changes in the chemical structure compared with that of the pristine D-T-ABPBI membrane. Generally, the PBI membranes used in VRFB operations are doped with sulfuric acid and exhibit excellent chemical resistance under acidic conditions [42,43]. Interestingly, when comparing the ATR-IR spectra of the active area of the tested membrane to those of the others, no peak shifts or changes that could be attributed to potential chemical structural changes were observed. The highly oxidizing VO_2_^+^ ions present in the positive electrolyte during the charge–discharge processes can degrade the polymer membrane during VRFB operation. To comprehensively investigate the changes in the chemical structure of the fabricated membranes, ^1^H-NMR analysis was performed (Figure 5d). Similar to the ATR-IR results, the ^1^H-NMR spectra of the tested membranes with an active area did not show any peak changes, such as peak shifts or the appearance of new peaks. This implies minimal structural changes in the active area of the D-T-ABPBI membranes induced by VO_2_^+^ as an oxidizing agent. We previously reported that ABPBI polymers show excellent chemical stability against highly oxidizing VO_2_^+^ ions when compared with other hydrocarbon-based polymers [24]. In the ex situ chemical stability test (1 M VO_2_^+^/5 M H_2_SO_4_), the D-T-ABPBI membrane exhibited chemical stability similar to the pristine ABPBI membrane against the oxidative VO_2_^+^, as shown in ESI (Figure S3). Moreover, both membranes demonstrated remarkable chemical stability comparable to that of Nafion 115, surpassing the o-PBI membrane. These results reaffirm that the ABPBI-based polymer membrane exhibits outstanding chemical stability under practical VRFB operating conditions.

## 4. Conclusions

A D-T-ABPBI membrane was successfully fabricated using the direct casting method. The WAXD patterns and dielectric constants of the membrane indicated that the free volume of the D-T-ABPBI membrane was larger than that of the S-T-ABPBI membrane fabricated using the common solution casting method. The increased free volume of the D-T-ABPBI membrane significantly enhanced its hydrophilic absorption capabilities. The membrane exhibited higher water absorption (*W_A_*) and water uptake (*W_W_*) when exposed to a 4 M H_2_SO_4_ solution than that of S-T-ABPBI (*W_A_* = 48.5% for D-T-ABPBI and 30.2% for S-T-ABPBI; *W_W_* = 47.0% for D-T-ABPBI and 22.7% for S-T-ABPBI). Consequently, the D-T-ABPBI membrane demonstrated a lower ASR of 0.98 Ωcm^2^, whereas S-T-ABPBI had an ASR of 1.75 Ωcm^2^. Owing to its high proton conductivity and low vanadium permeability, a VRFB cell assembled with the D-T-ABPBI membrane exhibited excellent performance, including excellent CE (>97%) and high VE and EE values (ranging from 93% to 70%). These values were comparable to those of Nafion 115. Furthermore, the D-T-ABPBI membrane demonstrated stable long-term operation over 300 charge–discharge cycles at 100 mAcm^−2^, indicating remarkable physicochemical stability in a highly oxidizing VO_2_^+^ electrolyte.

## Data Availability

The data presented in this study are available on request from the corresponding author.

## Supplementary Materials

The following supporting information can be downloaded at: https://www.mdpi.com/article/10.3390/polym15173577/s1, Figure S1: The mechanical strength of the D-T-ABPBI membrane, Figure S2: SEM images of the S-T-ABPBI membrane: (a) surface (b) cross-section, SEM image of the D-T-ABPBI membrane: (c) surface (d) cross-section, Figure S3: Ex situ chemical stability of prepared membranes: VO_2_^+^ concentration change over soaking time the test solution (1 M VO_2_^+^ /5 M H_2_SO_4_).
