# Supplementary material for: Fabrication of Tri-Directional Poly(2,5-benzimidazole) Membrane Using Direct Casting for Vanadium Redox Flow Battery
Source: Polymers (Basel). 2023 Aug 28;15(17):3577. doi: 10.3390/polym15173577 (PMC10490454; doi:10.3390/polym15173577)
Supplement: Supplementary file 1 [file polymers-15-03577-s001.zip › polymers-2558449-supplementary.pdf]

Supporting Information

Fabrication of tri-directional poly(2,5-benzimidazole) membrane using direct casting for vanadium redox flow battery

Jung-Kyu Jang*[a], and Tae-Ho Kim*[a]

[a.] Hydrogen Energy Research Center , Korea Research Institute of Chemical Technology (KRICT), Daejeon 34114, Republic of Korea;

*Email: jkjang@kreict.re.kr; thkim@krict.re.kr. Tel: +82-42-860-7379

Corresponding authors' e-mails: jkjang@kreict.re.kr and thkim@krict.re.kr

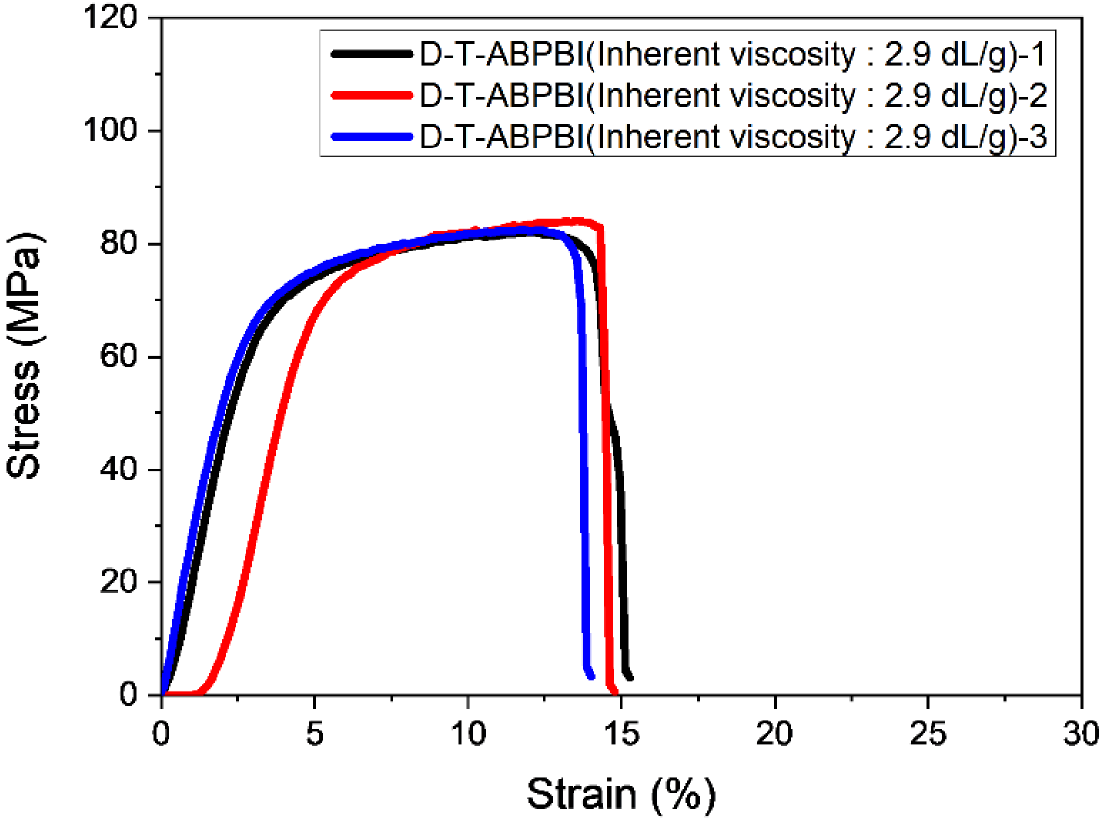

Figure S1. The mechanical strength of the D-T-ABPBI membrane

Characterization

The mechanical properties of the membranes were tested using a material testing

machine (Lloyd Instruments, LR5K) equipped with a 100 N load cell with a crosshead speed of 5 mm min^{-1} at RT. The prepared membranes measured 50 mm X 10 mm.

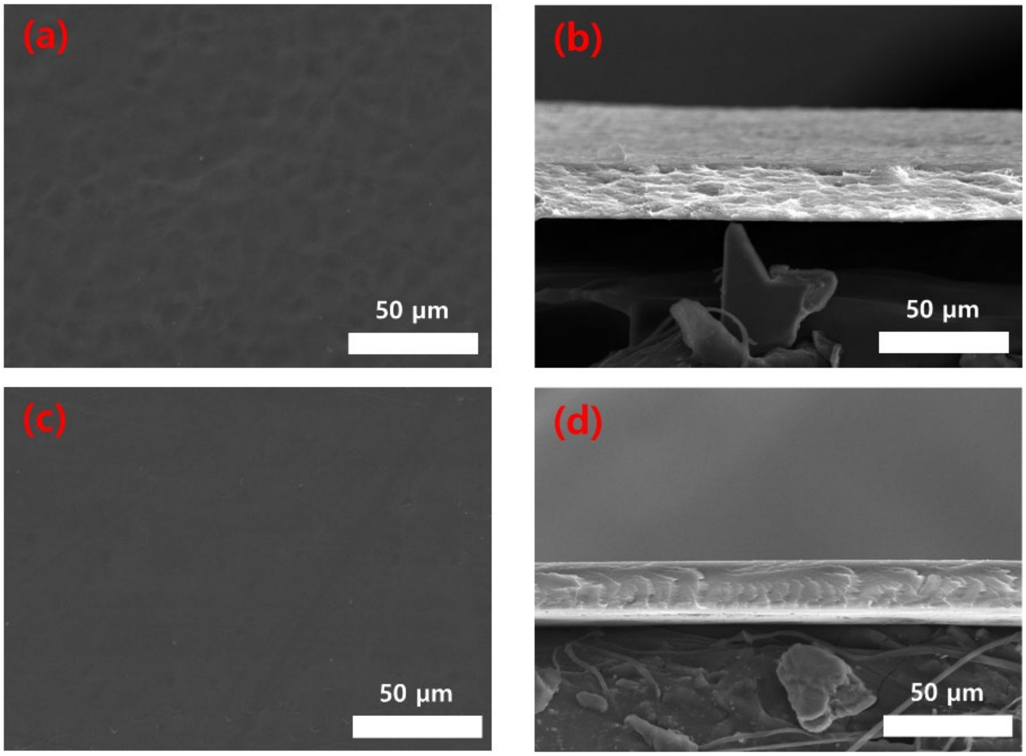

Figure S2. SEM images of the S-T-ABPBI membrane: (a) surface (b) cross-section, SEM image of the D-T-ABPBI membrane: (c) surface (d) cross-section.

Characterization

The surface and cross-sectional morphologies of the prepared membranes were observed using scanning electron microscopy (SEM, TESCAN Vega II LSU, Czech). The specimens were mounted on stubs with carbon tape and coated with thin platinum by sputter-coating.

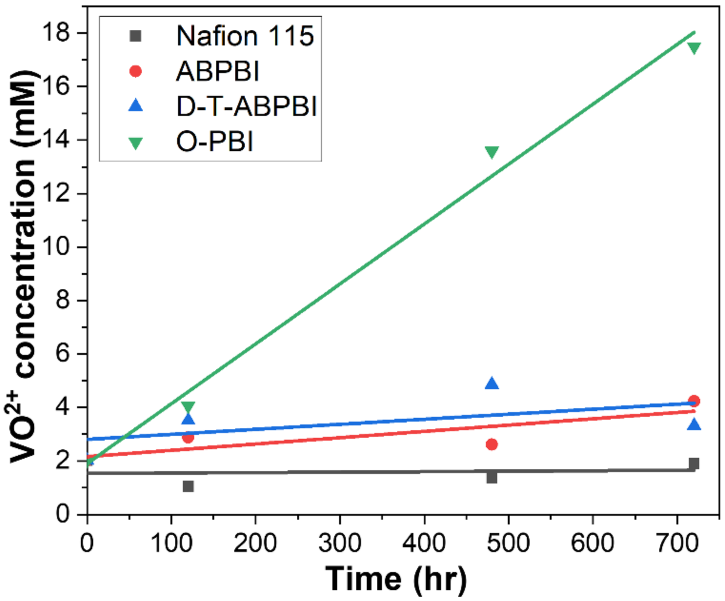

Figure S3. Ex situ chemical stability of prepared membranes: VO^{2+} concentration change over soaking time the test solution (1M VO_2^+ /5M H_2SO_4).

Characterization

The chemical stability of the membranes was conducted by an ex situ immersion test under a highly oxidizing 1 M VO_2^+solution. Nafion115, Poly(4,4'-diphenylether-5,5'-bibenzimidazole) (O-PBI), the pristine ABPBI membrane, and the D-T-ABPBI membrane were tested for comparison.

The prepared membranes were washed using isopropyl alcohol in a Soxhlet extractor for 2 days to remove any impurities. Subsequently, the membranes were dried under vacuum conditions at 80°C for 24 h before initiating the testing procedures.

The membrane samples were kept in polytetrafluoroethylene (PTFE) bottles containing 20 mL of 1 M VO_2^+/5 M H_2SO_4 solution at 40°C. Then, the change of absorbance at 760 nm as a function of immersion time was measured for each test solution using the UV−vis spectrometer. Note that, the measuring solutions were diluted to 0.1M VO_2^+ solution before the measurements were taken. The degree of degradation was assumed for prepared membrane from the increased VO^{2+}concentration of the test solution.
